# 

**Published:** 1875-02

**Authors:** 


### TABLE OF CONTENTS.

ORIGINAL COMMUNICATIONS.

Growth and Nutrition of the Body. By Joshua R. Graves, M. D.......... 65
Dropsy of the Amnion. By J. C. Bishop, M. D.......................... 72
Two Fatal Obstetrical Cases. By John Hockenhull, M. D................ 79
Hydrocele in a Female. By G. A. Baxter, M. D......................... 83
Lacerated Wound of Posterior Perinæum. By E. T. Henry, M. D.......... 84
Convulsions in Children. By Wm. T. Beall, M. D....................... 85

SELECTIONS.

Pathology and Treatment of Ovarian Diseases.......................... 87
Quinia as a Parturient............................................... 93

EXTRACTS AND GLEANINGS.

Progress of Surgery—96. Diseases of the Liver—97. Milk as a Remedy—98. Atropia in Ophthalmic Affections—99. Paralysis Caused by Worms; Chloroform as a Liniment; Bromide of Calcicum—100. Byrd on Cholera Infantum—101. The Suture of Tendons—102. Therapeutical Applications of Eucalyptus Globulus—103. Neuralgia; Tuberculosis not Incurable—104. Induction of Premature Labors; Galvano-Cautery; Nelson's Method of Resuscitation from Chloroform Narcosis—105. The Curability of Syphilis; The Use of Koumis; New Application of the Marsh Mallow—106. The Obstetric Swing; Influence of Anæsthetics upon the Sexual Impressions of Females; Diplopia and Ptosis with Syphilitic Paralysis and Muscular Atrophy—107. Croton Chloral; The Truth Comes Out—108. History of Cholera Summed Up; Heat from the Moon; Mammary Abscess—109. Cremation; Breathing in Rarefied Air; Camphor Water—110. Acute Bright's Disease; Treatment of Typhoid Fever; The Champion Chloroform-Tippler—111. To Keep Cider Sweet; Early Parturition; Subcutaneous Injection of Carbolic Acid in Traumatic Erysipelas—112. New Researches on Podophyllin; Injections of Cod-Liver Oil for Ascarides; To Promote Sound and Tranquil Sleep—113. Muriate of Ammonia in Bronchitis, Catarrhal Pneumonia, etc.; Lead in Soda-Water; Tonic Prescription—114. Chronic Poisoning with Chloral Hydrate; Gonorrhœa; Quinine and Morphine—115. Arsenic in Malignant Lympho-Sarcoma; Cardiac Œdema; A Good Cement—116. Cholera; Mucous Polypus Cured by Acetic Acid; Cod-Liver Oil Jelly—117. Unstopping a Deaf Ear; Camphor in Erysipelas; Removal of Nitric Acid Stains; Treatment of Spasmodic Asthma—118. Treatment of Salivation by Atropia; Enemata of Bromide of Potassium in Obstinate Vomiting; Treatment of Pneumonia and Bronchitis by Carbolic Acid; Aconite in Headache; Diabetes—119.

EDITORIAL AND MISCELLANEOUS.

To our Subscribers, - - - - - - - - 120
Our Advertising Department, - - - - - - 121
University Series of School Books; Query, - - - - - 122
The New Scriptures, - - - - - - - - 123
Communications; Morphia and Atropia Hypodermically Administered, - 126
A Shameful Demand by a Hospital Board; Book Notices, - - 127
List of Cash Payments to the RECORD, - - - - - - 128

